# The Institutional Life

**Published:** 1913-08-30

**Authors:** 


### THE INSTITUTIONAL LIFE.

Mr. H. G. Wells, viewing the great modern increase of mechanical transport, and consequently of mechanics to look after it, goes on to predict many political consequences from the formation of so large a homogeneous class of skilled manual workers. We will not indulge in vaticination on Mr. Wells' lines, but all the same it is worth pointing out that in the hospital sphere a somewhat parallel movement is taking place, if on a relatively (and, of course, actually) much smaller scale. As has been previously noted in these columns, there is a modern tendency to transfer treatment of sick persons from the home to the institution. This means more institutions, and these again more medical men to live and work in them; for over a certain number of beds in the case of chronic diseases, and always in the case of acute ones, resident medical supervision is a necessity. In the next decade there will be many more physicians in fever hospitals, Poor-Law infirmaries, mental hospitals, sanatoriums, and so forth than there were ten years ago. Beyond venturing the opinion that this will tend on the whole to raise the general level of professional efficiency, we will not touch on the main effects upon the profession itself; what we are now concerned with is the question, rather important practically, of the attractiveness of such a life.

Now a disgruntled person of experience might say that the life of a medical or lay resident consists in speaking to many different people every day, but knowing few of them well, and none of them anything but superficially; in parting largely with privacy and putting up with mainly fortuitous instead of stated leisure time; in too much preoccupation with the perforce rather petty customs of a world within a world; in some dread of the emotionally rather than rationally inspired decisions of a composite managing committee; in working rather for reputation than for remuneration; in short, in something not at all unlike what opponents of Socialism forecast as the probable lot of those living under that system. There is in all these charges some truth. Discipline forbids familiarity with coadjutors, who will nearly all be of junior standing; responsible managership, to be efficient, means both constant and devoted personal service, and the respecting of prejudices. No body of men whatever can, when assembled, escape the influence of crowd psychology; there is, to many minds, not much incentive about a fixed and rather moderate salary. The matter turns on temperament; one temperament will perceive the attractions where another will feel the above disadvantages.

First probably among the attractions we would put the possibility of maintaining high self-respect. It is quite possible for an institutional medical officer to know his subject to its depths. Now, in general practice this is possible to no man yet born. Nowadays general practice demands too much of anyone. Some or other of the deep-seated or rare diseases will baffle any general practitioner; baffle him even for their recognition and always for their treatment. And this breeds in him discontent—what a fine phrase-maker calls "the sulky sense of disadvantaged weakness." Coming to more mundane considerations, the absence of bad debts must be reckoned to the good, as also of the labour, waste of time, and wear and tear involved in travelling to patients, especially at night. With the sick man under good control at all times better results are obtainable. Indeed, the only disadvantage which there seems nothing to countervail is, we think, that of insecurity of tenure in those sorts of medical institutions which are more or less new founded and therefore unsettled in constitution. In general practice, even panel practice, it is the whole community by its units which really judges of a doctor. *Securus judicat orbis terrarum*; only the real evil-doer need fear the verdict. But a committee in session may judge hastily, with an unacknowledged lust to exercise its power of dismissal; moved, as has been said, by the psychology of the mob. Even this disadvantage will, however, tend to disappear with the passage of time and the resulting systematisation of the conditions of all resident officers' service. These conditions should be kept well in view by professional associations, for, as has been said, as time goes on they will become of more and more interest to institutional men.

